# Charles Wilson Peale as an Inventor of Porcelain Teeth

**Published:** 1889-06

**Authors:** 


Charles Wilson Peale as an Inventor of Porcelain Teeth:

1367 N. Gilmor, May 13, '89.

*F. J. S. Gorgas, M. D., D. D. S.,*

*Dear Doctor:*—Not knowing any one who would take more interest in any thing connected with the history of dentistry than yourself, I take the liberty of enclosing a copy of an original letter from that universal genius, Charles Wilson Peale in regard to his invention of porcelain teeth in 1823. The lady, Miss M. J. Peale, who kindly furnishes the copy, is a grand-daughter of the painter, and, I understand, inherits his genius in the use of the palette and brush. She is a distinguished painter, and as Peale was a Marylander, I take some pride in recalling the memory of one who added so much to an art and science, that has from the beginning been an especial product of the skill and genius of this State.

If you deem (as I hope you may) the letter worthy of publication, in some of your leading journals of dentistry, please preface it with the statement that Charles Wilson Peale was born at Chestertown, Kent county, Md., April 16, 1741. He was by trade a saddler, but followed at different times the occupations of watch and clock maker, silver-smith, preserver of animals, dentist and public lecturer. He received instruction in painting from Hesselins, a German, to whom he gave a saddle for the privilege of seeing him paint. He also made for himself a violin and a guitar, and in fact seemed an universal genius, and in all he attempted was self taught. In 1770 he went to London as a pupil of Benjamin West. Returning he first established himself at Annapolis, but afterwards removed to Philadelphia, where he became a distinguished portrait-painter. His benevolence was equal to his genius, for he gave his services in aid of Dr Benjamin Rush in the terrible yellow fever epidemic that prevailed in Philadelphia, 1798. He served as captain in the battles of Trenton and Germantown. He originated a museum, the largest of its kind in the United United States, in Philadelphia in 1785, and a similar one in Baltimore in 1813 on Holliday street, where the old City Council stood. This museum was afterwards removed to north-west corner of Calvert and Baltimore streets, till bought by Mr. P. T. Barnum from Mr Edmund Peale in 1845, when the building was transformed into a theatre, but still called the Museum. Mr. C. W. Peale died in 1827, aged 85, leaving a son, Rembrandt, who inherited his genius and was an artist of great merit.

I only cite these facts to refresh your memory on what is doubtless not so well known by the younger generation of the career of this extraordinary man. You can select what you please, or reject all if you deem proper. But if the letter be published, please send me a copy of the Journal where it appears, and also have one directed to Miss M. J. Peale, 1411 Walnut street, Philadelphia. When you have used the letter, please return it and oblige.

Yours very respectfully,

JNO. R. QUINAN.

{ Philadelphia, April 10th, 1889.
{ 1411 Walnut St.

*Dr. John R Quinan :—*

So long a time has elapsed since you wrote, desiring information and date of grand-father's making porcelain teeth. I could not give you the exact date, and owing to my uncle, Mr. Titian Peale's death and his wife having softening of the brain, his effects could not be touched until her death this winter, and I now can give you the date from his own letter, although it is probably too late for your work. I send a copy of his letter which makes it perfectly authentic. I may find something about his work at the time the yellow fever was here. I know that he worked with Dr. Rush, but I may find it in his own hand-writing. I regret exceedingly that so much time has passed, but I could not be sure as the older members of the family have all passed away.

Respectfully yours,

M. J. PEALE.

I will copy what he writes :

Philadelphia, July 26th, 1826.

"*Dear Sir :*—It is only of late, not more than three months, since I have determined to make porcelain teeth for the public. Upwards of three years I have made them for my particular friends, relations and myself, but knowing or rather believing that this labour being of considerable more importance than that of painting portraits, or in short any other employment that I can do, I am preparing furnaces to extend the work, after which I shall be willing to aid dentists with porcelain teeth in several forms, but, of late, I have found it difficult to procure platina, except at a terrible price; but it can be had in Paris and London, and from thence will procure it. At the last time I sent to London for platina, the merchant was requested to send a few specimens of platina teeth for my inspection, but the merchant finding that they demanded the exorbitant price of 35f. per tooth, he did not send them, requesting further orders. It was fortunate he did not send them, as I suppose a sufficient profit may be made at one dollar a tooth.

'I know Mr. Gournsey's method of making teeth, and approve of it more than of other artists. When I have advanced in my work, will write to you again, with respect,

Your friend,

C. W. PEALE.

To THOMAS W. PARSONS, ESQ., Boston."

[One of the letters which grand-father copied in his own hand writing.—M. J. PEALE.]

